# Survival and prognostic factors in patients undergoing pulmonary metastasectomy for lung metastases from retroperitoneal sarcoma

**DOI:** 10.1186/s12957-022-02552-y

**Published:** 2022-04-08

**Authors:** Fumiaki Takatsu, Hiromasa Yamamoto, Yasuaki Tomioka, Shin Tanaka, Kazuhiko Shien, Ken Suzawa, Kentaroh Miyoshi, Shinji Otani, Mikio Okazaki, Seiichiro Sugimoto, Masaomi Yamane, Katsuhito Takahashi, Shinichi Toyooka

**Affiliations:** 1 Department of General Thoracic Surgery and Breast and Endocrinological Surgery, Okayama University Graduate School of Medicine, Dentistry and Pharmaceutical Sciences, Okayama, 700-8558 Japan; 2 Department of Thoracic Surgery, Okayama University Hospital, 2-5-1 Shikata-cho, Kita-ku, Okayama, 700-8558 Japan; 3 Organ Transplant Center, Okayama University Hospital, Okayama, 700-8558 Japan; 4 Center for Innovative Clinical Medicine, Okayama University Hospital, Okayama, 700-8558 Japan; 5 Center for Sarcoma Multidisciplinary Treatment, Department of Sarcoma Medicine, Kameda Medical Center, Kamogawa, Chiba 296-8602 Japan

**Keywords:** Retroperitoneal sarcoma, Lung metastasis, Metastasectomy

## Abstract

**Background:**

Soft-tissue sarcomas are rare malignancies that consist of many different histologic subtypes and arise in various locations in the body. In patients with lung metastases from retroperitoneal sarcomas, the long-term outcomes and prognostic factors are unknown. This study is a retrospective review of patients undergoing pulmonary metastasectomy for retroperitoneal sarcoma metastases at one institution, with the purpose of determining prognostic factors and clinical outcomes.

**Methods:**

This is a single-center, retrospective cohort study of patients undergoing pulmonary metastasectomy for lung metastases from various sarcomas at Okayama University Hospital from January 2006 to December 2018. The Kaplan-Meier method and log-rank test were used for the analyses, and cut-off values of continuous variables were determined by a receiver operating characteristic curve analysis.

**Results:**

Twenty-four patients underwent the first pulmonary metastasectomy for lung metastases from retroperitoneal sarcoma in our hospital. Leiomyosarcoma was the most common histologic subtype of retroperitoneal sarcoma (79.2%, *n* = 19). Median overall survival was 49.9 months, and the 3-year and 5-year survival rates after the first pulmonary metastasectomy were 62.5% and 26.4% respectively. In univariate analysis, age ≥56 years, disease-free interval < 15 months, and size of metastasis (≥ 27 mm) were associated with poor survival.

**Conclusion:**

Pulmonary metastasectomy can be considered as an effective management strategy in retroperitoneal sarcoma patients with lung metastases in appropriately selected cases, just as it is for other sarcomas.

## Background

Retroperitoneal sarcoma (RPS) is a rare malignancy with an overall incidence of 0.5–1/100,000 [1]. The retroperitoneum represents the second-most common site of primary mesenchymal malignancies; the most common are those arising from the lower extremities [2]. One-third of malignant tumors in the retroperitoneum are sarcomas, and 15% of soft-tissue sarcomas occur in the retroperitoneum [3].

Several studies have reported that RPS carries a poorer prognosis than sarcomas on other anatomic locations, with 5-year overall survival (OS) of 39–68% even after complete resection of the primary lesion. Due to the anatomic location of these tumors [4–9], RPS frequently presents with non-specific symptoms until the tumor has reached a significant size. Most patients with RPS present with abdominal swelling, early satiety, and abdominal discomfort [10]. They often have very large tumors at the time of diagnosis; the median weight of resected primary RPS tumors is 4.0 kilograms [11]. Recurrence patterns vary by histologic type, with lung and liver being the most common sites of distant recurrence. Although outcomes for metastatic RPS are also reported to be poor [12, 13], metastasectomy could be considered as one of the treatment options for disease control, as long as the possibility of long-term survival remains. This is particularly true because effective chemotherapeutic or molecular-targeted drugs have yet to be developed.

Recently, we reported on pulmonary metastasectomy (PM) for lung metastases from various sarcomas using our database of patients undergoing PM between 2006 and 2015, in which we showed the neutrophil-to-lymphocyte ratio (NLR) to be an independent prognostic factor [14]. We advocated the prognostic scoring system (Sarcoma Lung Metastasis Score), which is based on preoperative prognostic factors [15]. While a number of studies, including ours, have demonstrated the long-term outcomes and prognostic factors of PM for lung metastases from various sarcomas, no reports are available specifically for PM in RPS patients with lung metastases.

In this study, we updated the database we used for our prior paper by extending the inclusion criteria to patients who underwent PM from the original end date of December 2015 by 3 years, to December 2018. We reviewed the survival data again, focusing on determining the clinicopathological characteristics and prognostic factors of the patients undergoing PM for lung metastases from RPS, as well as clarifying the significance of PM in the clinical management of these patients.

## Patients and methods

### Patient selection

**Fig. 1:**
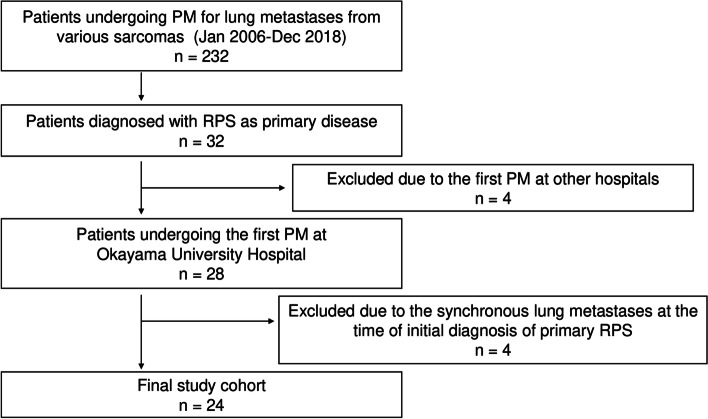
Flow diagram of this study. PM, pulmonary metastasectomy; RPS, retroperitoneal sarcoma

We maintain a database of the patients undergoing PM for lung metastases from various primary sarcomas in Okayama University Hospital [14, 15]. Using our updated database, we conducted a retrospective review of a total of 232 patients who underwent PM between January 2006 and December 2018. Of the 232, 32 patients were diagnosed with RPS as the primary sarcoma. Four patients who had undergone their first PM in other hospitals and another four patients who developed synchronous lung metastases at the time of initial diagnosis of primary RPS were excluded, resulting in a final cohort of 24 patients (Fig. 1).

Patients were diagnosed with RPS by histological examination of the primary lesion, and the presence of lung metastasis was confirmed by histological examination of the surgical specimens from PM. Patients who underwent PM for lung metastases from RPS met the following criteria [14–16]: (a) the primary tumor was completely resected; (b) all metastatic disease was completely resectable or controllable with local therapies; (c) the patients had a suitable performance status; (d) the planned procedure entailed acceptable anticipated complications, and (e) the patient’s respiratory function was sufficient to tolerate planned pulmonary resection.

Follow-up for the patients was generally done every 6–12 months after PM and included physical examination, blood tests, and chest X-ray or CT.

This retrospective study protocol (No. K1612-033) was approved by the Ethics Committee of Okayama University Graduate School of Medicine, Dentistry and Pharmaceutical Sciences and Okayama University Hospital, and written informed consent from each patient was waived.

### Data collection

The variables in the model included age, sex, histologic findings, whether or not chemotherapy was used for the primary tumor, disease-free interval (DFI), extent of lung metastases at the first PM, presence of local recurrence and/or extrapulmonary metastasis with or before the diagnosis of lung metastasis, the use of chemotherapy for lung metastasis, surgical approach [open thoracotomy, mini-thoracotomy, or video-assisted thoracoscopic surgery (VATS)], type of resection, size of the largest resected lesion, number of resected lesions, completeness of resection, frequency of PM (repeated surgery), and NLR immediately before the first and the most recent PM. The surgical approach was defined by skin incision size: open thoracotomy (> 8 cm), mini-thoracotomy (> 3 cm, ≤ 8 cm), and VATS (≤ 3 cm). Complete resection was defined as the removal of all lesions that were known at the time of the first PM, via a one- or two-stage operation. DFI was defined as the time interval between removal of the primary retroperitoneal sarcoma and the first diagnosis of lung metastasis. OS was calculated as the time interval from the first PM until death or the last recorded follow-up.

### Statistical analysis

All statistical analyses were performed with EZR version 1.42 (Saitama Medical Center, Jichi Medical University, Saitama, Japan), a graphical user interface for R (The R Foundation for Statistical Computing, Vienna, Austria) [17]. GraphPad Prism 9.0 software (San Diego, CA, USA) was used for graphic display.

Quantitative variables were expressed as median values. OS was calculated according to the Kaplan-Meier method, and differences among the groups were assessed by the log-rank test. The receiver operating characteristic (ROC) curve for 3-year mortality was obtained to calculate optimal cut-off values to differentiate quantitative variables. We defined *p* < 0.05 as the threshold for statistical significance.

## Results

### Patient characteristics

The characteristics of the 24 patients are shown in Table 1. Median age at the time of the first PM was 56 years (range: 36–70 years). In fact, 83.3% of the patients (*n* = 20) were female. Leiomyosarcoma was the most common histological subtype (79.2%, (*n* = 19), followed by dedifferentiated liposarcoma (12.5%, (*n* = 3). The median DFI was 16.8 months (range: 1.6–139.5 months). The median NLR values immediately before the first PM and the most recent PM were 2.32 (range: 1.12–7.52) and 2.30 (range: 0.83–7.52) respectively.

**Table 1:** Characteristics of the patients undergoing the first PM for lung metastases from RPS (*n* = 24)

Variables	Results
Age (years)	
Median (range)	56 (36–70)
Sex	
Male	4 (16.7%)
Female	20 (83.3%)
Histological subtypes of primary RPS	
Leiomyosarcoma	19 (79.2%)
Dedifferentiated liposarcoma	3 (12.5%)
Others	2 (8.3%)
Disease-free interval (months)	
Median (range)	16.8 (1.6–139.5)
Extent of lung metastasis	
Unilateral	8 (33.3%)
Bilateral	16 (66.7%)
Local recurrence and/or extrapulmonary metastasis with or before lung metastasis	
Yes	11 (45.8%)
No	13 (54.2%)
Preoperative chemotherapy for lung metastasis	
Yes	9 (37.5%)
No	15 (62.5%)
NLR immediately before the first PM	
Median (range)	2.32 (1.12–7.52)
NLR immediately before the most recent PM	
Median (range)	2.30 (0.83–7.52)

*PM* Pulmonary metastasectomy, *RPS* Retroperitoneal sarcoma, *NLR* Neutrophil-to-lymphocyte ratio

### Surgical interventions

Characteristics of the surgical procedures are shown in Table 2. At the time of the first PM, the majority of patients (70.8%, *n* = 17) had a wedge resection. In 6 patients (25.0%) PM was performed by anatomic segmentectomy, and by lobectomy in 1 patient (4.2%). The median size of the largest resected tumor was 18 mm (range: 6–75 mm). A total of 15 patients (62.5%) underwent repeated PM, and the median frequency of PM was 2 (range: 1–8). The median number of resected tumors per intervention was 5 (range: 1–21), and the total number of resected tumors per patient in the study period was 6 (range: 1–42). Complete (R0) resection was accomplished in 20 patients (83.3%).

**Table 2:** Characteristics of the surgical procedures (*n* = 24)

Variables	Results
Surgical approach at the first PM	
Open	7 (29.2%)
Mini-thoracotomy	13 (54.2%)
VATS	4 (16.7%)
Type of resection at the first PM	
Lobectomy	1 (4.2%)
Segmentectomy	6 (25.0%)
Wedge resection	17 (70.8%)
Size of the largest resected tumor (mm)	
Median (range)	18 (6–75)
Maximum number of resected tumors per intervention	
Median (range)	5 (1–21)
Total number of resected tumors in the study period	
Median (range)	6 (1–42)
1-5	7 (29.2%)
6-10	10 (41.7%)
11 ≤	7 (29.2%)
Complete resection	
Yes	20 (83.3%)
No	4 (16.7%)
Repeated resection	
Yes	15 (62.5%)
No	9 (37.5%)
Frequency of PM	
Median (range)	2 (1–8)

*VATS* Video-assisted thoracoscopic surgery, *PM* Pulmonary metastasectomy

### Survival analyses

**Fig. 2:**
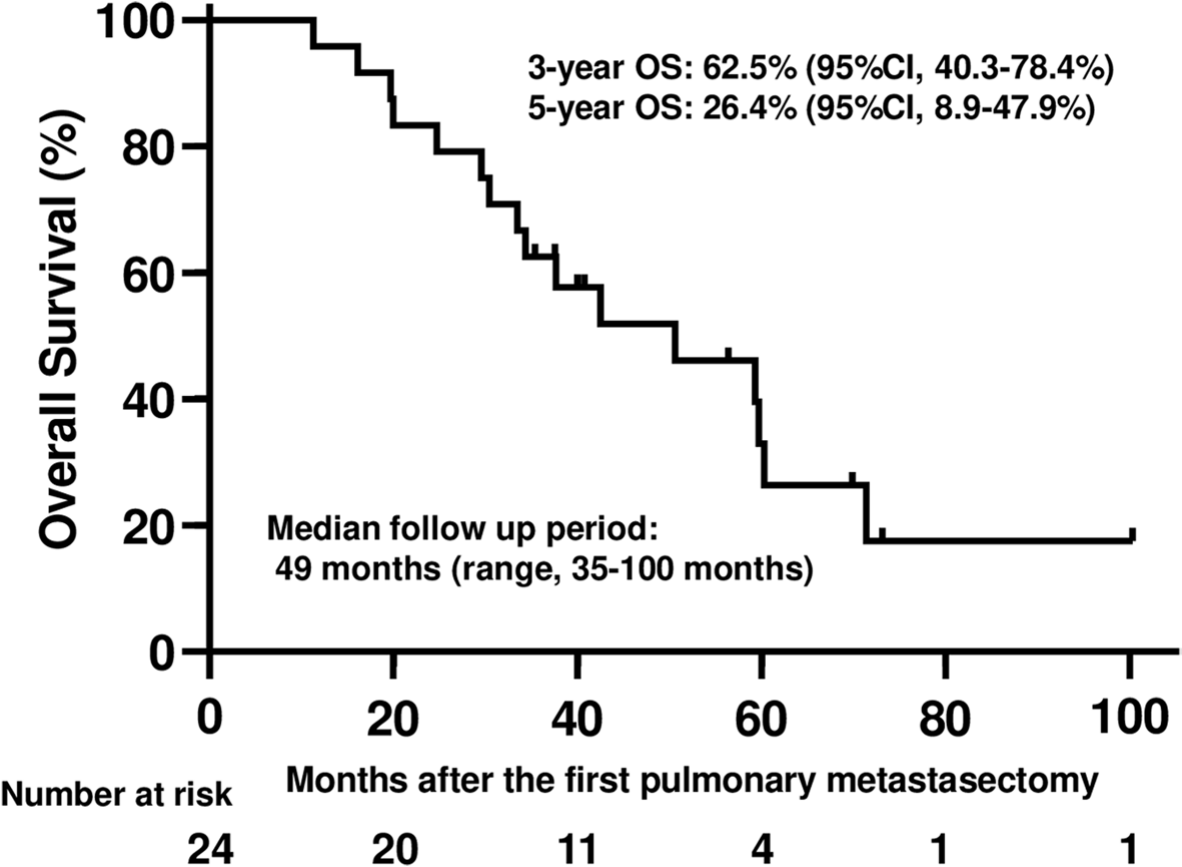
OS of the patients with lung metastases from retroperitoneal sarcoma after the first pulmonary metastasectomy

The median follow-up time for survivors in this study was 49 months (range: 35–100 months). Median OS was 49.9 months (95% confidence interval [CI] = 30.0–59.4 months), and 3-year and 5-year survival rates were 62.5% and 26.4% respectively (Fig. 2). In the univariable analysis, age ≥ 56 (*p* < 0.001), DFI (< 15 months, *p* = 0.04), and the size of the largest resected tumor (≥ 27 mm, *p* = 0.04) were identified as significant negative prognostic factors (Table 3). The ROC curve determined cut-off values for quantitative variables as follows: DFI 15 months (AUC 0.67), largest resected tumor size 27 mm (AUC 0.63), NLR immediately before primary PM 1.92 (AUC 0.50), and NLR immediately before the most recent PM 2.48 (AUC 0.62). Since the cut-off values for age and total number of resected tumors were biased, only these variables were calculated with the median as the cut-off. Due to the small number of events in this study, the multivariable analysis was not performed.

**Table 3:** Univariate analysis (log-rank test) for overall survival (*n* = 24)

Variables	n	3-year OS	95% CI	*P* value
Age (years)				< 0.001
< 56	11	90.9%	50.8–98.7	
≥ 56	13	38.5%	14.1–62.8	
Sex				0.28
Male	4	50.0%	5.8–84.5	
Female	20	65.0%	40.3–81.5	
Histological subtypes				0.01
Leiomyosarcoma	19	73.7%	47.9–88.1	
Dedifferentiated liposarcoma	3	NA	NA	
Others	2	NA	NA	
Disease-free interval (months)				0.04
< 15 months	9	44.4%	13.6–71.9	
≥ 15 months	15	73.3%	43.6–89.1	
Extent of lung metastases				0.2
Unilateral	8	62.5%	22.9–86.1	
Bilateral	16	62.5%	34.9–81.1	
Preoperative chemotherapy for lung metastases				0.84
Yes	9	55.6%	20.4–80.5	
No	15	66.7%	37.5–84.6	
NLR before the first PM				0.8
< 1.92	6	83.3%	27.3–97.5	
≥ 1.92	18	55.6%	30.5–74.8	
NLR before the most recent PM				0.41
< 2.48	15	60.0%	31.8–79.7	
≥ 2.48	9	66.7%	28.2–87.8	
Surgical approach				0.16
Open	7	42.9%	9.8–73.4	
Mini-thoracotomy	13	69.2%	37.3–87.2	
VATS	4	75.0%	12.8–96.1	
Type of resection				0.31
Lobectomy	1	100.0%	NA	
Segmentectomy	6	50.0%	11.1–80.4	
Wedge resection	17	64.7%	37.7–82.3	
Size of the largest resected tumor				0.04
< 27mm	17	76.5%	48.8–90.4	
≥ 27mm	7	28.6%	4.1–61.2	
Total number of resected tumors				0.6
< 7	13	61.5%	30.8–81.8	
≥ 7	11	63.6%	29.7–84.5	
Complete resection				0.17
Yes	20	70.0%	45.1–85.3	
No	4	25.0%	0.9–66.5	
Repeated resection				0.41
Yes	15	66.7%	37.5–84.6	
No	9	55.6%	20.4–80.5	

*RPS* Retroperitoneal sarcoma, *NLR* Neutrophil-to-lymphocyte ratio, *PM* Pulmonary metastasectomy

## Discussion

RPS has varying clinical courses depending on histologic grade and subtype. The most common histologic subtypes of primary RPS are liposarcoma (41%) and leiomyosarcoma (28%) [3], while leiomyosarcoma (71%) is the most frequent subtype of lung metastases of RPS followed by liposarcoma (18%). The tendency of the tumor to metastasize differs among the histological subtypes. These findings are supported by a previous study suggesting that the histological subtype leiomyosarcoma was an independent risk factor for developing distant metastases, with a 5-year metastasis risk of 41% compared with an 18% risk for the overall series [5].

The lungs are the most common site of metastases in patients with sarcoma [18]. However, there is no evidence that this prognostic improvement of the patients with lung metastases from sarcoma is truly attributable entirely to metastasectomy and it is usual in surgery to rely mainly on evidence from case series due to the absence of control data [19]. No randomized controlled trials regarding PM have been reported except for the study in colorectal cancer [20]. That randomized control trial of PM in colorectal cancer is nested within a prospective observational study of about 500 patients [21]. In those two reports, the patients who did not undergo PM had better survival than was assumed, and survival in the metastasectomy group was comparable with the many single-arm follow-up studies. Taken together, the authors in those two reports concluded that most of the apparent survival differences can be accounted for by the highly selective use of PM in patients with known favorable characteristics. A number of studies regarding the use of PM for soft-tissue sarcomas have reported a 5-year OS range from 11 to 71% [22]. Since all the reports concern soft-tissue sarcoma in general, with the proportion of RPS at 0–19%, our study is the first to characterize the patients with lung metastases of RPS who underwent PM. For the survival after PM, 3-year and 5-year OS were 62.5% and 26.4% respectively, roughly equal to other sarcoma subtypes. The reported 5-year survival rate after PM in colorectal cancer patients is 20–68% [23], which is consistent with the 26.4% 5-year OS rate in our cohort. Therefore, surgical treatment seems to be acceptable in RPS patients with lung metastases if they are appropriately selected. Although PM itself cannot be proven to contribute to improving the prognosis, it would benefit the patients if the compressed lung can re-expand and become functional again by removing lesions acting as space occupiers, for instance. Regarding the repeated metastasectomies, we previously discussed the importance of preserving lung parenchyma as much as possible when performing PM, as more chances of PM for the local treatment of lung metastases from sarcomas can be advantageous to the sarcoma patients [14]. Repeated surgery can be beneficial for the patients with the second lung metastases after PM, although it may be difficult if lung metastases recur as numerously multiple tumors. Several prognostic features associated with long-term survival in sarcoma patients undergoing PM have been identified, including complete resection of all metastases, DFI, advanced stage, and original size of primary tumors, synchronous detection of metastases, age, largest size of the metastatic tumors, and the number of lesions [3, 24–26]. In our study, older age, shorter DFI, and larger size of lung metastases were the significant factors for poor prognosis. Although we previously found that NLR is an independent prognostic factor [14], there was no significant difference in the current study. The limited number of patients might have affected these results.

There are several important limitations in our study. First, we had a small sample size due to the rarity of RPS, limiting the power of our statistical findings. Second, our data were derived from a single-center retrospective survey. In addition, the survival rates were evaluated in patients undergoing surgery with curative intent, introducing the inevitable selection bias. Systemic reviews and multicenter series can help further clarify appropriate patient selection and the benefit of PM in these patients.

## Conclusions

Using a single-institution database, we have identified the characteristics and prognostic factors for patients with lung metastases from RPS undergoing PM. Older age, shorter DFI, and larger size of lung metastases were associated with poor survival. In selected cases, PM can continue to be considered an effective management strategy in RPS patients with lung metastases, just as it is in other sarcomas.

## Data Availability

The datasets used and/or analyzed during the current study are available from the corresponding author on reasonable request.

## Abbreviations

RPS: Retroperitoneal sarcoma
OS: Overall survival
PM: Pulmonary metastasectomy
NLR: Neutrophil-to-lymphocyte ratio
DFI: Disease-free interval
VATS: Video-assisted thoracoscopic surgery
